# A125 TANDEM STUDY DESIGN IS LESS LIKELY TO DEMONSTRATE IMPROVED ADENOMA DETECTION RATE THAN PARALLEL STUDY DESIGN IN THE ASSESSMENT OF ARTIFICIAL INTELLIGENCE-ASSISTED COLONOSCOPY

**DOI:** 10.1093/jcag/gwac036.125

**Published:** 2023-03-07

**Authors:** M C Lee, T Jeyalingam, C H Parker, L W Liu

**Affiliations:** Division of Gastroenterology and Hepatology, Department of Medicine, University Health Network, University of Toronto, Toronto, Canada

## Abstract

**Background:**

Randomized controlled trials have reported that artificial intelligence (AI) improves adenoma detection rate (ADR). Different methodologies, namely parallel and tandem study designs, have been employed to evaluate the efficacy of AI-assisted colonoscopy in randomized controlled trials. In systematic reviews and meta-analyses, a pooled effect that includes both study designs have been reported. However, it is unclear whether there are inconsistencies in the reported results of these two designs.

**Purpose:**

To determine if there are differences in ADR using AI-aided technologies during colonoscopy between parallel and tandem study designs

**Method:**

A systematic search of Ovid MEDLINE (1946 to October 2022) and EMBASE (1947 to October 2022) for randomized controlled trials comparing AI-assisted colonoscopy with routine high-definition colonoscopy in polyp detection was conducted. Reference lists of systematic reviews were searched for additional studies. The publications were divided based on trial design: parallel vs. tandem. Analysis was conducted using Review Manager 5.4.1 using a random effects model.

**Result(s):**

The search identified 540 articles. After screening the title and abstract for relevance, 19 randomized controlled trials involving a total of 14 657 patients were included for full-text review. Fourteen were parallel studies (14 136 patients) and 5 were tandem studies (521 patients). ADR was reported in 17 studies, and there was overall improvement in ADR with AI-assisted colonoscopy (risk ratio [RR] 1.33, 95% CI 1.22-1.44; p<.0001). Based on a separate pooled analyses of 13 parallel studies and 4 tandem studies, ADR significantly improved with AI assistance compared to routine colonoscopy, regardless of study design (RR 1.35, 95% CI 1.24-1.47 and p<.0001; RR 1.15, 95% CI 1.03-1.28; p=0.02, respectively). A significant increase in ADR with AI assistance were found in 84.6% (11/13) of parallel design studies, but in only 25% (1/4) of tandem studies.

**Image:**

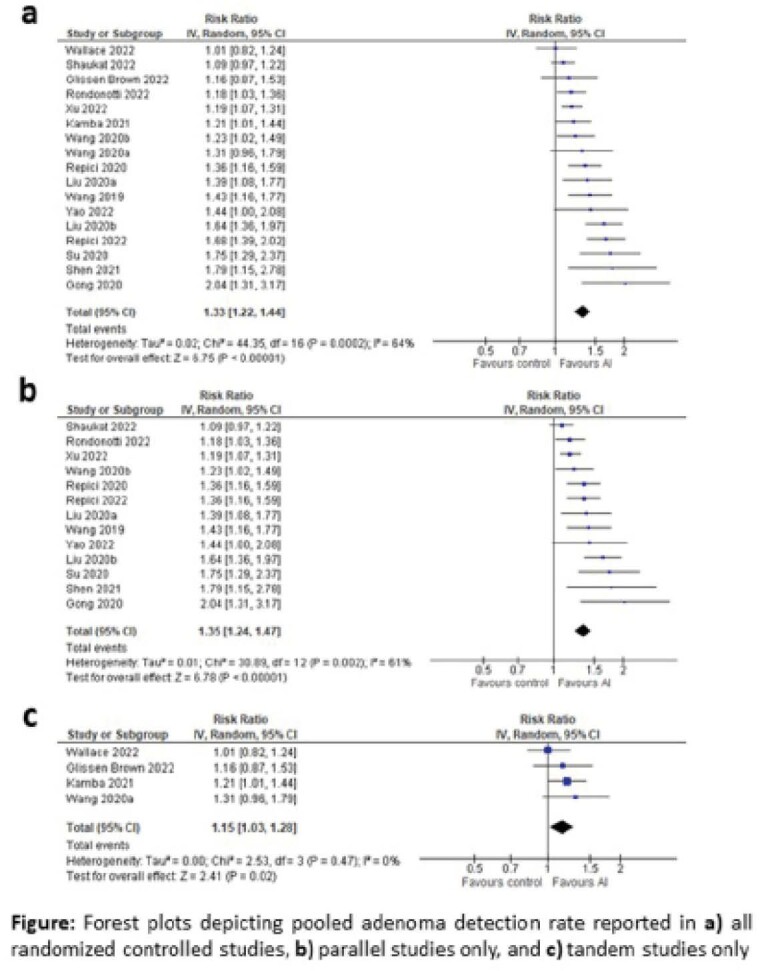

**Conclusion(s):**

AI assistance during colonoscopy significantly increased ADR in both parallel and tandem studies; however, improvement in ADR was less likely to be seen in tandem compared to parallel studies. It remains unclear why this difference exists, but some hypotheses include smaller sample sizes in the tandem studies, significant heterogeneity in the tandem design, and differences in operator bias depending on study design. Better understanding the differences in these study designs will inform future studies of new endoscopic technologies.

**Disclosure of Interest:**

None Declared

